# Antiproteinase 3 Positive Eosinophilic Granulomatosis with Polyangiitis Presenting with Heart Failure and Intraventricular Thrombosis

**DOI:** 10.1155/2017/2908185

**Published:** 2017-01-29

**Authors:** Dan Zhu, Yiming Luo, Xiangyuan Liu, Lingyun Zu

**Affiliations:** ^1^Department of Cardiology, Peking University Third Hospital, Key Laboratory of Cardiovascular Molecular Biology and Regulatory Peptides, Ministry of Health, Beijing, China; ^2^Department of Medicine, Mount Sinai St. Luke's and Mount Sinai West Hospitals, Icahn School of Medicine at Mount Sinai, New York, NY, USA; ^3^Department of Rheumatology and Immunology, Peking University Third Hospital, Beijing, China

## Abstract

Eosinophilic granulomatosis with polyangiitis (EGPA) is a rare systemic vasculitis commonly with cardiac complications. We describe a case of anti-PR3 ANCA-positive EGPA complicated by congestive heart failure and intraventricular thrombosis. Interestingly, the thrombus was resolved rapidly with steroid and cyclophosphamide in the setting of interrupted anticoagulation. To the best of our knowledge, we report the first case of anti-PR3 positive EGPA with extensive cardiac involvement. Our patient had overlapping features with previously studied ANCA-positive and ANCA-negative EGPA cases. We also hypothesize that the thrombogenic potential of eosinophils may play a central role in thrombogenesis in EGPA and aggressive immunosuppressive therapy remains the cornerstone of treatment, and the addition of anticoagulation therapy in the setting of thrombus formation and also very high risk of bleeding needs to be considered cautiously.

## 1. Introduction

Eosinophilic granulomatosis with polyangiitis (EGPA), formerly named Churg-Strauss syndrome, is a rare systemic vasculitis of small- and medium-sized vessels, with heart involvement being a common complication. We present a case of a 20-year-old male with antiproteinase 3 (PR3) antineutrophil cytoplasmic antibodies (ANCA) positive EGPA complicated by severe cardiomyopathy and intraventricular thrombosis, which responded robustly to aggressive immunosuppressive therapy with successful thrombolysis in the setting of interrupted anticoagulation therapy.

## 2. Case Presentation

**Figure 1:**
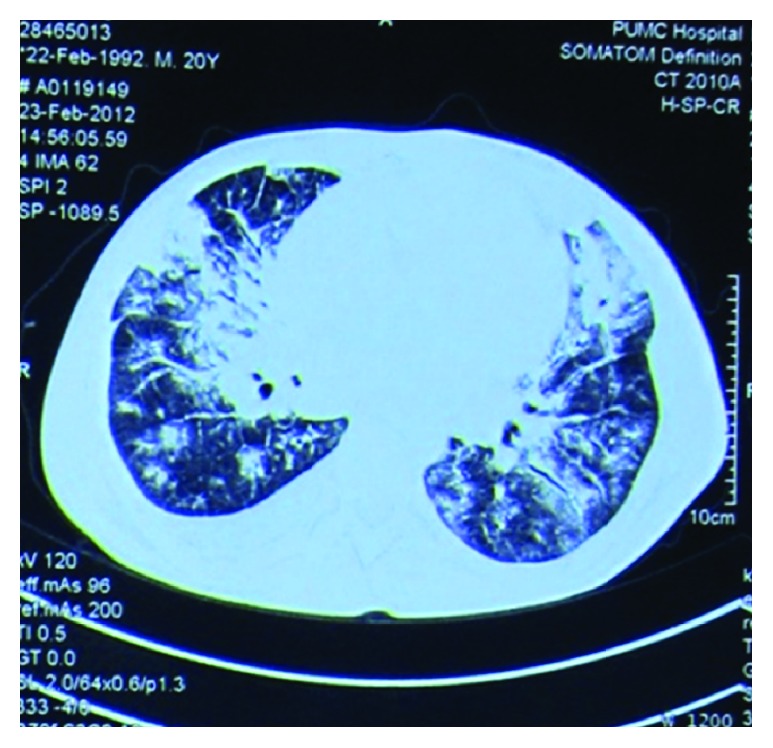
Chest CT. It shows bilateral patchy ground-glass opacities and subpleural nodules.

**Figure 2:**
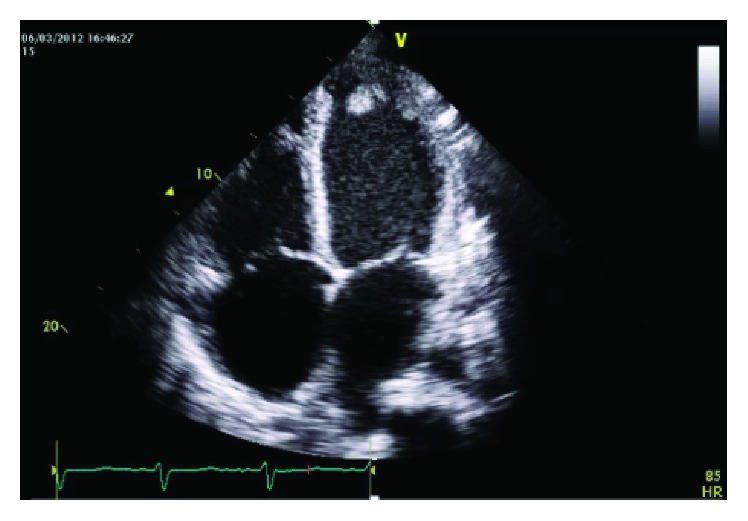
Transthoracic Echocardiogram (TTE). A left ventricular apical intramural thrombus can be visualized.

A 20-year-old male with a 6-month history of chronic cough, progressive exertional dyspnea, and recurrent epistaxis was admitted for worsening dyspnea. He also complained of right lower extremity burning pain and numbness. On physical examination, he had generalized lymphadenopathy, S3 gallop, bilateral pulmonary crackles, hepatomegaly, and peripheral edema. No skin rash was noted. Neurological examination revealed flattening of the nasolabial fold, decreased sensation over the right lower extremity, and generalized hypoactive tendon reflex. Complete blood count showed elevated eosinophil count of 7390 n/*μ*L, which contributed to 28.1% of leukocytes. Erythrocyte sedimentation rate was 69 mm/h. N-terminal of the prohormone brain natriuretic peptide (NT-proBNP) was 27523 pg/mL upon admission. Liver function tests showed alanine transaminase 1441 u/L, total bilirubin 29.0 *μ*mol/L, and albumin 23.9 g/L. Urinalysis and creatinine were normal. Immunological workup showed positive ANCA with anti-PR3 level of 91 IU/mL but negative for antimyeloperoxidase (MPO), as well as an IgE level of above 2500 IU/mL. Antinuclear antibodies and rheumatoid factor were negative. Electrocardiogram demonstrated nonspecific ST-T wave changes. Serial chest X-ray in the hospital showed migratory infiltrative changes compared to the one from outside the hospital three weeks before. Chest CT scan showed bilateral ground-glass opacities and multiple subpleural nodules (Figure 1). Transthoracic Echocardiogram (TTE) demonstrated four-chamber dilatation, decreased wall motion, and apical intramural thrombus, with left ventricular ejection fraction (LVEF) of 26% (Figure 2). Nasal polyps were detected by rhinoscopy and excisional biopsy showed small amount of intraepithelial eosinophilic infiltration and subepithelial amyloid deposition. Bone marrow biopsy was negative for leukemia and also with negative FIP1L1-PDGFRA and PDGFRB fusion genes. Based on the above clinical, laboratory, and imaging findings, a diagnosis of EGPA was made.

The patient was treated with pulse methylprednisolone 500 mg intravenously daily for 5 days and then gradually tapered to 60 mg orally daily and cyclophosphamide 400 mg intravenously once, followed by 100 mg orally daily. Warfarin was also started with enoxaparin bridging therapy. His heart failure was managed by aggressive diuresis. His hospital course was complicated by upper gastrointestinal tract bleeding at day 4 so anticoagulants were discontinued. Repeated TTE at day 7 revealed complete resolution of intraventricular thrombus; at the same time, his eosinophil count was lowered to 0. He was discharged after significant symptomatic improvement and his LV EF was back to 34%.

## 3. Discussion

We described a case of a young male who presented with severe cardiomyopathy with intraventricular thrombus, clinical peripheral neuropathy, nasal polyps, pulmonary nodules, hepatitis, eosinophilia, and anti-PR3 ANCA positive. The diagnostic discrimination between EGPA and hypereosinophilic syndrome (HSE) can be challenging, especially in the absence of histologically proven vasculitis. The absence of FIP1L1-PDGFRA and PDGFRB gene fusion and unremarkable bone marrow biopsy ruled out most clonal forms of HSE. Positive ANCA can be used as a surrogate marker for vasculitis [1]. And our case fits into the American College of Rheumatology 1990 classification criteria [2]. Therefore, we consider our case a diagnosis EGPA. In clinical practice, a histological diagnosis may not always be possible and the classic pathological triad of necrotizing vasculitis, eosinophilic infiltration, and extravascular granuloma rarely coexisted in any one patient [3]. At the meantime, anti-PR3 ANCA is very rare in EGPA [4] and false positive ANCA, although also rare, is possible [5]. Thus, our case calls for a both accurate and practical classification and diagnostic criteria to aid clinical judgement and better understand the utility of surrogate markers in the setting of atypical presentations. Currently, a multinational observational study is underway for this challenge [6].

Due to the heterogeneity of EGPA, efforts have been made to identify its subtypes. Unlike granulomatosis with polyangiitis (GPA) and microscopic polyangiitis (MPA), only 40% of EGPA is ANCA-positive. Studies [4, 7] have suggested a phenotypic distinction between ANCA-positive and ANCA-negative EGPA, with the former associated with renal involvement, peripheral neuropathy, and biopsy-proven vasculitis and the latter associated with higher amount of eosinophilia and cardiac involvement. However, those studies mainly included patients with anti-MPO ANCA. To the best of our knowledge, we report the first case of anti-PR3 ANCA-positive EGPA with extensive cardiac involvement. And our patient had overlapping features among previously studied ANCA-positive and ANCA-negative EGPA cases [4, 7]. Due to its rarity, how to incorporate anti-PR3 ANCA positivity into subcategorization of EGPA warrants further investigations.

Cardiac involvement is well documented in patients with EGPA, with incidence ranging from 17% to 92% depending on the diagnostic modality and accounting for almost 50% mortality in EGPA. Cardiac involvement is also included in the Five-Factor Score (FFS) which predicts a worse outcome and an indication for adjunct cytotoxic treatment with cyclophosphamide in addition to systemic glucocorticoid [8]. Although this recommendation was based on expert opinion [3], Miszalski-Jamka et al. [9] found that lack of noncorticosteroid immunosuppression is an independent determinant of cardiac involvement in EGPA and the extent of myocardial damage is associated with insufficient duration of noncorticosteroid immunosuppression.

Intraventricular thrombosis has been reported in patients with EGPA and was presumably caused by eosinophilic endocarditis [10]. The underlying pathophysiology of cardiac thrombus in patients with EGPA is unknown. It has been shown that eosinophils may play an important role in thrombosis especially in hypereosinophilic disorders, involving enhanced tissue factor expression [11] and generating prothrombotic fibrin structure [12]. We demonstrated complete resolution of intraventricular thrombus within one week following aggressive immunosuppressive therapy in the setting of interrupted anticoagulation therapy. It is possible that treating eosinophilia removed the driving factor of thrombogenesis and subsequently led to rapid thrombolysis. Complete resolution of intraventricular thrombus by aggressive immunosuppression without anticoagulation in systemic vasculitis has been reported in literature [13, 14], including one case of EGPA [14]. Although there is no consensus over this particular setting, the selection and duration of anticoagulation therapy need to be considered more cautiously in patients with very high risk of bleeding or the occurrence of clinical bleeding is uncertain but cannot be ruled out.

In conclusion, we present a case of anti-PR3 ANCA-positive EPGA with extensive cardiac involvement, which responded rapidly with aggressive immunosuppressive therapy. The atypical clinical presentation calls for more accurate and practical classification and diagnostic criteria. The biological and clinical significance of anti-PR 3 ANCA in EGPA is not clear. And the optimal duration of anticoagulation therapy for thrombosis in systemic vasculitis treated with aggressive immunosuppressive therapy warrants more investigations.
